# Gene Coexpression Network and Module Analysis across 52 Human Tissues

**DOI:** 10.1155/2020/6782046

**Published:** 2020-05-02

**Authors:** Binsheng He, Junlin Xu, Yingxiang Tian, Bo Liao, Jidong Lang, Huixin Lin, Xiaofei Mo, Qingqing Lu, Geng Tian, Pingping Bing

**Affiliations:** ^1^Academician Workstation, Changsha Medical University, Changsha 410219, China; ^2^College of Computer Science and Electronic Engineering, Hunan University, Changsha, Hunan 410082, China; ^3^School of Mathematics and Statistics, Hainan Normal University, Haikou 570100, China; ^4^Geneis Beijing Co., Ltd., Beijing 100102, China

## Abstract

Gene coexpression analysis is widely used to infer gene modules associated with diseases and other clinical traits. However, a systematic view and comparison of gene coexpression networks and modules across a cohort of tissues are more or less ignored. In this study, we first construct gene coexpression networks and modules of 52 GTEx tissues and cell lines. The network modules are enriched in many tissue-common functions like organelle membrane and tissue-specific functions. We then study the correlation of tissues from the network point of view. As a result, the network modules of most tissues are significantly correlated, indicating a general similar network pattern across tissues. However, the level of similarity among the tissues is different. The tissues closing in a physical location seem to be more similar in their coexpression networks. For example, the two adjacent tissues fallopian tube and bladder have the highest Fisher's exact test *p* value 8.54*E*-291 among all tissue pairs. It is known that immune-associated modules are frequently identified in coexperssion modules. In this study, we found immune modules in many tissues like liver, kidney cortex, lung, uterus, adipose subcutaneous, and adipose visceral omentum. However, not all tissues have immune-associated modules, for example, brain cerebellum. Finally, by the clique analysis, we identify the largest clique of modules, in which the genes in each module are significantly overlapped with those in other modules. As a result, we are able to find a clique of size 40 (out of 52 tissues), indicating a strong correlation of modules across tissues. It is not surprising that the 40 modules are most commonly enriched in immune-related functions.

## 1. Introduction

With the development of next-generation sequencing technologies, there are more and more sequencing data available, which provides a great opportunity to unravel the biological mechanisms behind them using bioinformatics and machine learning tools [1]. As one of the most efficient bioinformatics methods, biological networks are frequently used to visualize the interactions among molecules [2]. Gene coexpression networks are one type of important biological networks, in which each node denotes a gene and each edge denotes a gene coexpession between two genes. A gene is coexpressed with the other one if there are some correlations between the expression profiles of the two genes across the sample set, which might be caused by gene regulation and other biological mechanisms. Similar to other networks, modularity is a very important feature of the gene coexpression network. A gene expression module is a set of genes, in which each pair of genes are highly coexpressed. Previous studies have suggested that genes in the same module tend to perform similar functions corporately and thus, a gene module might indicate some critical function [3]. The key driver genes in a module are usually highly associated with disease progression and patients' survival, which are usually used as diagnostic biomarkers and drug targets [4].

There are a lot of studies on gene coexpression networks. Zhang and Horvath developed weighted gene coexpression network analysis (WGCNA) [5], an algorithm to construct the gene coexpression network and modules from expression of single genes. WGCNA first calculates the absolute value of the Pearson correlation between expression vectors of two genes, which is then powered to a certain value. The topological overlap matrix (TOM) is then calculated to consider the similarity of adjacent genes, and finally, a hierarchical clustering algorithm is applied to obtain gene modules. The algorithm was implemented in R [6] and widely used in many studies. For example, Ghazalpour et al. applied the gene coexpression network to characterize genes associated with the mouse weight [7]. Zhao explored gene coexpression modules associated with lung adenocarcinoma by using WGCNA [8]. Similarly, Lv et al. identified lung cancer-related modules based on the coexpression network [9]. Li et al. identified key pathways and genes in the dynamic progression of hepatocellular cancer (HCC) through WGCNA and key driver analyses [10]. In addition, Gargalovic et al. identified inflammatory gene modules based on human endothelial cell responses to oxidized lipids, which turn out to be a gold module in many WGCNA analyses [11]. Miller et al. examined the divergence of human and mouse brain transcriptome, by which they identified pathways associated with Alzheimer's disease [12]. Finally, it worth's mentioning that gene coexpression networks were also used to study protein interactions [13], brain damage [14], or the effect of certain drugs on damaged tissues [15].

However, most of the studies only focus on one coexpression network or compare two gene coexpression networks under two conditions (e.g., disease versus normal or human versus mouse). A systematic comparison of coexpression networks and modules across a cohort of human tissues is more or less ignored. Comparing tissue-specific coexpression networks is essential to evaluate tissue heterogeneity, which is critical for tissue-specific disease studies and drug design.

In this study, we construct 52 tissue or cell line-specific coexpression networks and their modules from the genotype-tissue expression (GTEx) project. We then annotate the modules in each coexpression network. By comparing the pair-wise enrichment of modules among networks, we infer tissue correlations from the network point of view. It is known that immune-associated modules are gold modules for WGCNA analyses. We thus study the enrichment of immune function in modules for all 52 networks. Finally, we perform a maximal clique analyses to retrieve modules conserved across many tissues and also tissue-specific modules. This study provides a network view of human tissues.

## 2. Materials and Methods

### 2.1. Data Source and Preprocessing

We downloaded the gene expression data in fpkm format of 52 tissues and cell lines from the GTEx Official website (https://gtexportal.org), which include Adipose_Subcutaneous, Adipose_Visceral_(Omentum), Adrenal_Gland, Artery_Aorta, Artery_Coronary, Artery_Tibial, Brain_Amygdala, Brain_Anterior_cingulate_cortex_(BA24), Brain_Caudate_(basal_ganglia), Brain_Cerebellar_Hemisphere, Brain_Cerebellum, Brain_Cortex, Brain_Frontal_Cortex_(BA9), Brain_Hippocampus, Brain_Hypothalamus, Brain_Nucleus_accumbens_(basal_ganglia), Brain_Putamen_(basal_ganglia), Brain_Spinal_cord_(cervical_c-1), Breast_Mammary_Tissue, Cells_EBV-transformed_lymphocytes, Cells_Transformed_fibroblasts, Colon_Sigmoid, Colon_Transverse, Esophagus_Gastroesophageal_Junction, Esophagus_Mucosa, Esophagus_Muscularis, Heart_Atrial_Appendage, Heart_Left_Ventricle, Liver, Lung, Muscle_Skeletal, Nerve_Tibial, Ovary, Pancreas, Pituitary, Prostate, Skin_Not_Sun_Exposed_(Suprapubic), Skin_Sun_Exposed_(Lower_leg), Small_Intestine_Terminal_Ileum, Spleen, Stomach, Testis, Thyroid, Uterus, Vagina, Whole_Blood, and so on, and the number of samples for each tissue varied from 71 to around 500. The readers are referred to the GTEx website for the detailed information of tissues and samples (https://gtexportal.org). The genes in each tissue or cell lines were overlapped, and the profiles for the overlapping genes were kept for each tissue and cell line. As suggested by the GTEx consortium, the expression profile for each sample is then mapped to a standard normal distribution according to the rank of gene expression across samples. As a result, the gene expression profile for each gene satisfies a normal distribution, and thus, the Pearson correlation is suitable for calculating the correlation between genes. The normalized gene expression data was then used to construct coexpression networks and modules.

### 2.2. Construction of Gene Coexpression Network

#### 2.2.1. Prerequisites for Network Construction

— In the WGCNA algorithm, the elements in the gene coexpression similarity matrix are the absolute values of correlation between expressions of two genes, raised up to a given power. The power is a user-defined parameter determined usually by the scare-free property of the resulted coexpression network. Let *k* be a variable denoting the possible degrees of a node, varying from 1 to the maximum node degree *K*, and *p*(*k*) be the probability of a node to be of degree *k*; that is, the number of nodes with degree *k* divided by the number of all nodes. The Pearson correlation between log(*k*) with *k* from 1 to *K* and log(*p*(*k*)) with k from 1 to *K* is used to evaluate the scare-free of a network. A higher correlation indicates that the network is more scare-free. Usually, we require the correlation to be greater than 0.8, by which to select the WGCNA parameter power.

#### 2.2.2. Major Steps for Network Construction

     

**Step 1 . Choose a similarity measure.**

There are many ways to measure similarity, such as the Pearson correlation coefficient, which is one of the most common and widely studied measures [16]. So we can choose the Pearson correlation to calculate the similarity between expression vectors of two genes. The similarity of gene coexpression network can be expressed by a similarity matrix. The element of matrix is the correlation coefficient between each pair of genes. The gene network is an undirected graph, so the calculation of a similarity value should be absolute. The similarity formula is as follows:

(1) $$\begin{array}{c} S_{\text{mn}=}\left|\text{cor}\left(m,n\right)\right|. \end{array}$$

**Step 2 . Define adjacency functions.**

The process of defining adjacency function is to determine threshold *β*. There are two main ways to determine threshold, hard threshold and soft threshold. Soft threshold is based on network topology [17], and the network is weighted network, which is more robust than unweighted network. The adjacency function used in the WGCNA method is the power exponential adjacency function. So we can get adjacent matrix *Amn*. The element of matrix, *amn*, is the connection strengths between each pair of genes.

That is to say, for any pair of genes, in order to get the connection strengths *Amn*, the weighting coefficient *β* is subjected to a power exponential operation on the correlation coefficient *Smn*. The formula is as follows:

(2) $$\begin{array}{c} \;a_{mn}=\text{power}\left(S_{mn},\beta \right). \end{array}$$

**Step 3 . Computation of Dissimilarity.**

The degree of difference between genes is the basis for constructing gene modules. A simple method of calculating the degree of difference is that the weighted correlation coefficient in the adjacency matrix can be directly used as the similarity, and then, the similarity is subtracted from 1 to be the dissimilarity. However, it is proposed that when the module is obtained from the clustering of gene expression profiles, the dissimilarity based on TOM similarity is better. The topological overlap matrix is used to calculate the similarity of adjacent genes. The topological overlap of two nodes *ω*_*mn*_ reflects their relative interconnectedness.

(3) $$\begin{array}{c} \omega_{mn}=\frac{{\text{l}}_{mn}+{\text{a}}_{mn}}{\min\left\{k_{m}+k_{n}\right\}+1-a_{mn}}. \end{array}$$

where *a*_*mn*_ is the element of adjacent matrix, *l*_*mn*_ is the sum of the product of the adjacent coefficients of the nodes connected to the genes *m* and *n*, *l*_*mn*_ = ∑*a*_*mu*_*a*_*un*_, . And *k*_*m*_ and *k*_*n*_ represent the sum of the adjacent coefficients of the nodes to which the genes *m* or *n* are individually connected.

Therefore, using 1 minus the TOM similarity will result in a better module. Just like the software design concept, the module which satisfies low cohesion and high coupling is an ideal one. One of the TOM similarities is based on the TOM similarity measure on the topological structure [18], and the other is the proposed extended topology overlap matrix [19].

**Step 4 . Cluster analysis to get the module.**

After the identification of genes is determined, a hierarchical clustering tree is constructed. The construction of clustering trees has two algorithms: static cut tree and dynamic cut tree [20]. But the static cut tree algorithm is clustered by defining a fixed height, and the accuracy of the method to identify the cluster is not high. The dynamic cut tree algorithm is based on the branch shape of the tree diagram, which can be used to mine more information in the gene modules that cannot be detected by the static algorithm. More importantly, the gene network identified by the dynamic algorithm is consistent with the results of previous biological experiments [21]. So in the WGCNA algorithm, the dynamic algorithm is used to get the modules.

### 2.3. The Fisher's Exact Test

The *p* value of the Fisher's exact test is used to judge the null hypothesis. The null hypothesis is usually assumed to have no relationship between the two objects, so if the p value is less than 0.05, the null hypothesis can be overturned, and the opposite hypothesis is derived. In the experiment, the *p* value of every module in one tissue is calculated with each module in other tissues. When the result *p* value is less than 0.05, the two modules are related.

### 2.4. The Clique Analysis

The maximum clique problem is one of the most canonical problems in computer science and graph theory. Given a network, it identifies the largest complete graph; that is, the graph with each pair of nodes is connected. Theoretically, this problem is NP-complete. However, there are a few heuristic algorithms like Genetic Algorithm, Simulated Annealing, and the Tabu Algorithm, which were implemented in several R packages. In this study, we used the maximum clique function in the R package igraph to identify the maximum clique in a given network.

### 2.5. The Gene Ontology Analysis

Gene ontology is an ontology library that contains three group classes: cellular components, molecular function, and biological process. Gene ontology analysis is aimed at identifying the GO terms enriched for a given gene set, which was usually done by the hypergeometry test. In the experiment, GO analysis was carried out from two aspects: one is the tissue GO analysis and the other one is the analysis of modules generated from the tissue.

### 2.6. Threshold Selection for WGCNA

**Figure 1:**
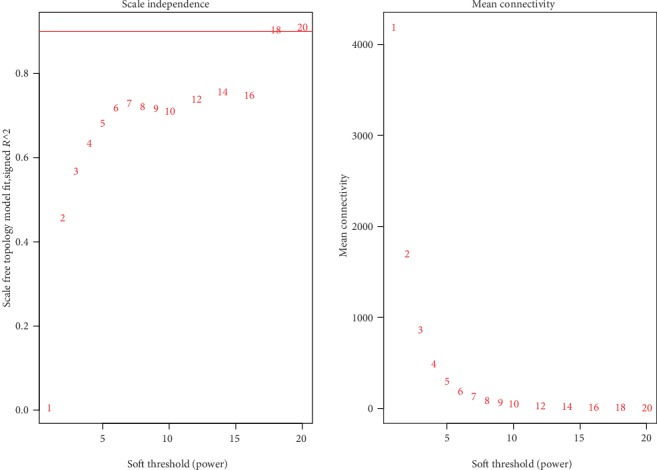
Threshold selection for an example tissue. (a) Description of the different power and the R2. (b) Description of the different power and the mean connectivity.

Figure 1 below shows the threshold selection graph for one of the tissues adipose visceral omentum. The abscissas of the left and right graphs in Figure 1 both mean different threshold selections. The ordinate on the left graph indicates the square of the correlation coefficient between log (*k*) and log (*p*(*k*)) in the corresponding generated network. The ordinate on the right graph shows the average connectivity of the nodes in the network. Ideally, if the correlation coefficient on the left graph reaches 0.8, the value on the right graph will be high, and vice versa. For this tissue, the threshold is 18 to ensure that the correlation coefficient is greater than or equal to 0.8. However, from the right graph, it seems that the curve is flat after a threshold value of 6. In practice, the WGCNA website usually suggests 6 as the threshold, which usually achieves reasonably good results [20]. To make a fair comparison across all tissues, we selected the threshold to be 6 for all tissues and cell lines.

## 3. Results

### 3.1. Gene Coexpression Modules Are Significantly Enriched in Gene Ontology Terms

Take the spleen tissue as an example. A network graph was created by WGCNA for the spleen tissue, with a total of 18,648 genes and 20 modules. Analysis of GO in spleen tissue shows that spleen's more prominent function is mainly the basic components of cells, such as GO:0005829~cytosol, GO:0005783~endoplasmic reticulum, and GO:0031090~organelle membrane, and cell life cycle, such as GO:0022402~cell cycle process, as well as cellular metabolic activities, such as GO:0010557~positive regulation of the macromolecule biosynthetic process and GO: 0009891~positive regulation of the biosynthetic process. Besides that, there are some functions related to gene expression, such as GO:0032553~ribonucleotide binding, GO:0032555~purine ribonucleotide binding, and GO:0006357~regulation of transcription from RNA polymerase II promoter.  Table 1 is the example of some functions.

Take the dark red module and dark turquoise module of spleen tissue as an example to analyze their functions.

**Figure 2:**
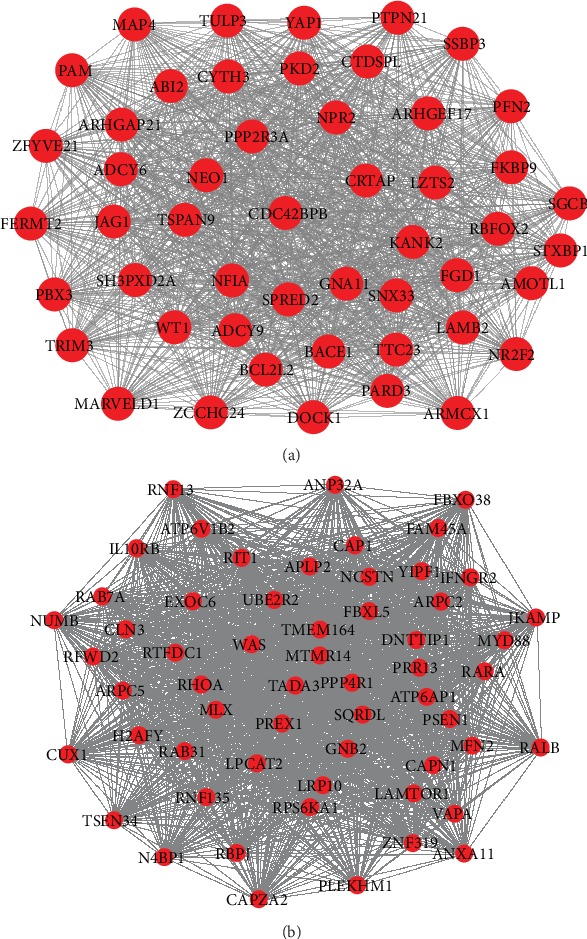
The two graphs are the module networks. (a) The network of darkred module of spleen. (b) The network of darkturquoise module of spleen.

Figure 2 is the network of the dark red module and the dark turquoise module of spleen tissue. Both of the two graphs are composed of a number of nodes, which has a large degree.

From the GO analysis of the dark red module, it can be seen that the module mainly reflects the correlation between the spleen and other human tissues and systems, such as GO:0001501~skeletal system development, GO:0001568~blood vessel development, GO:0001944~vasculature development, GO:0007423~sensory organ development, and GO:0007517~muscle organ development. It can be seen that the spleen is a special organ that is closely related to many distant organs and plays a role with them. The importance of the spleen may be manifested in the connection with these organs [22].

From the GO analysis of the dark turquoise module, it can be seen that the module mainly reflects the function of spleen immunity, such as GO:0006955~immune response, GO:0006952~defense response, GO:0006954~inflammatory response, and GO:0045087~innate immune response. All in all, the spleen is also mainly related to immune activity. Because the spleen is the largest immune organ of human body, accounting for 25% of the total lymphoid tissue in the whole body and contains a large number of lymphocytes and macrophages, which is the center of cellular immunity and human immunity.

**Figure 3:**
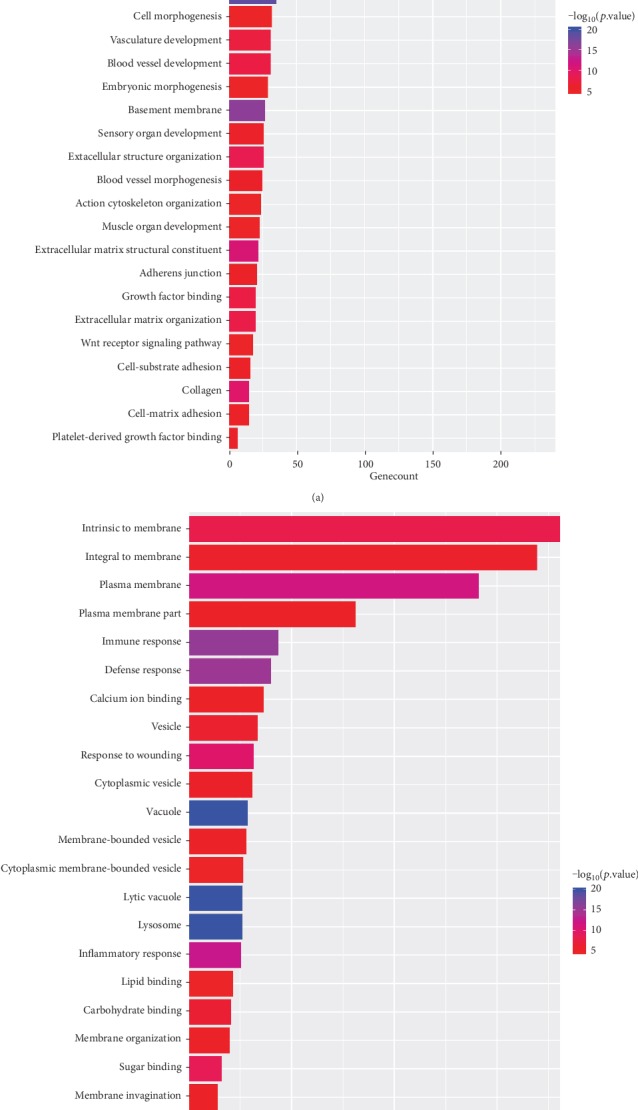
The two graphs are the GO results of module networks of spleen. (a) The GO result of dark red module. (b) The GO result of dark turquoise module.

Figure 3 shows the enriched GO analysis of the two modules.

### 3.2. The Relationship of all Tissues

Fisher's exact test was performed on all tissues to measure correlations and found that all tissues were related in pairs. This shows that although the structure and function of different human body tissues are different, they are not isolated when they carry out various life activities. They closely cooperate with each other. For example, when we exercise vigorously, not only the activity of the skeletal muscles of the whole body is strengthened but also the breathing is deepened and quickened. So more oxygen can be inhaled, and more carbon dioxide can be exhaled. At the same time, the heartbeat is also accelerated, which promotes faster circulation of blood, delivers more nutrients and oxygen to skeletal muscles, and carries more waste. Therefore, the various human tissue systems are coordinated activities and fully demonstrate that the human body is a unified whole.

**Figure 4:**
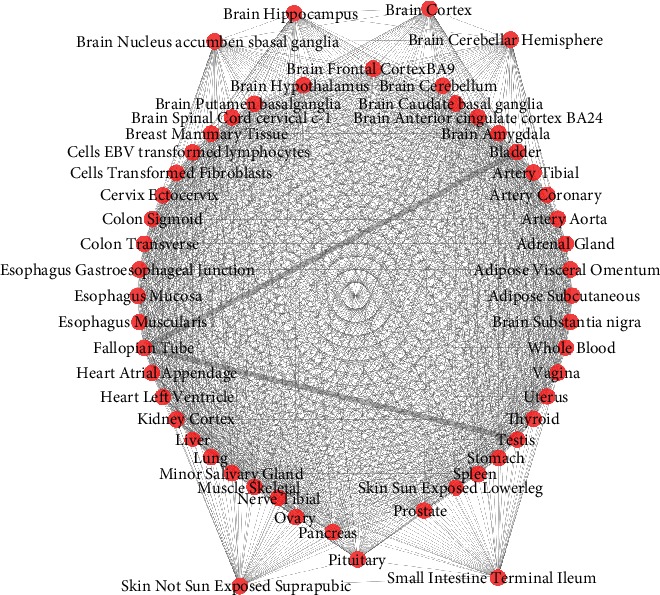
Tissue relationship diagram.

Figure 4 shows the relationship among tissues. The two bold lines show the closed tissues and the most different tissues.

### 3.3. Two Closed Tissue Analysis

The two closest tissues were found to be the fallopian tube and bladder. The *p* value of both of them reached 8.54*E*-291. This is mainly because the two tissues have close location and similar structure.

The Fallopian tube is a female-specific reproductive organ located on the upper edge of the broad ligament of the uterus. For women, the bladder and uterus are in close proximity. Therefore, the position of the Fallopian tube and bladder is generally very close. And the Fallopian tube and bladder both belong to the hollow organ type, and the structure is very similar. From the GO of the two tissues, the functions are also very similar. Enriched functional coincidence items reached 83, mainly concentrated in the basic components of cells, organelles, and cell membrane; mitotic cycle, cell death, apoptosis, and other cell life cycle; and macromolecular synthesis process regulation and metabolic process regulation.

### 3.4. Analysis of Tissue Functions

GO analysis of tissues with the DAVID online tool (https://david-d.ncifcrf.gov) revealed that there were 19 GO items in each tissue, which mainly focused on the basic components of the cell, such as GO:0031090~organelle membrane and GO:0005829~cytosol. In addition, there are base synthesis activities, such as GO:0030554~adenyl nucleotide binding, GO:0017076~purine nucleotide binding, GO: 0030554~adenyl nucleotide binding, and the metabolism of phosphorus, GO:0006793~phosphorus metabolic process, because phosphorus is an important component of genetic material nucleic acids. And it is also an important component of adenosine triphosphate.

Immunity or inflammation-related functions are basically enriched in each tissue. This is mainly because lymphocytes exists everywhere in the body, and lymphocyte is an important part of the immune system, and lymphocyte travels throughout the body through blood and lymph, from one lymphoid organ or lymphoid tissue to another lymphoid organ or lymphoid tissue. So that the scattered lymphoid organs and lymphoid tissue can be linked into a functional whole.

However, not every tissue is highly enriched in with immune-related functions. Immune cell function will behave differently because of the different organs or tissues located in. The experiment shows that some of the modules of the tissue of liver, kidney cortex, lung, uterus, adipose subcutaneous, and adipose visceral omentum are highly enriched with immune-related functions. Studies have shown that there are local immune responses in these tissues because of the presence of specialized tissue-resident immune-related cells [23]. The liver has superior innate immunity because it has natural killer cells, which are important lymphocytes of the innate immune system [24]. The kidney has a large number of immune cells, dendritic cells, and macrophages, which are not only related to immune response but also the renal tissue injury and subsequent reparative reactions [25]. Macrophages in adipose play an important role in the immune regulation of metabolism and are the necessary effector cells to coordinate metabolic inflammation [26] and metabolic process regulation.

### 3.5. Largest Clique Analysis

Correlation analysis is also performed for all modules, and then, the largest complete sub graph of 40 modules is screened out in the all modules diagram with *p* value less than 0.05. The study of tissue correlation shows that the tissues are related to each other, so 40 modules belong to different tissues, but they are also related to each other. At the same time, the 40 modules have similar biological functions. Most of them focused on immune inflammation-related regulation and metabolic processes and cell components. The graph below shows the network of the largest clique.

**Figure 5:**
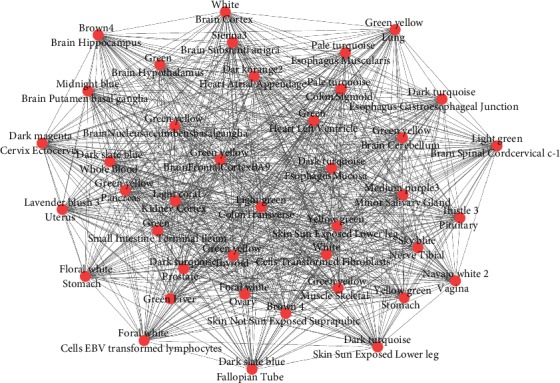
The network of largest clique.

Figure 5 shows the relationship among tissues. The two bold lines show the closed tissues and the most different tissues.

Using DAVID to perform GO analysis on the modules in the largest clique, it was found that the immune function is highly enriched in the lavenderblush3 module of Uterus, the green module of small intestine terminal ileum, and the yellow green module of Stomach, and the green yellow module of Lung.

An important immune cell in the uterus is MC (mast cell), located in the uterine wall. MC can produce 5-HT. 5-HT is not only an important neurotransmitter but also an important immunoregulatory factor. It has both immunosuppressive and immunopromoting effects. 5-HT has a regulatory effect on delayed type hypersensitivity, natural killing activity of NK cells, lymphocyte activity, and macrophage function. It is an important component of the neuroendocrine immune network. 5-HT directly acts on T lymphocyte, B lymphocyte, or other immune cells through various types of receptors on the different surfaces of immune cells [27].  Table 2 shows the enriched immunity for uterus.

In the terminal ileum of the small intestine, there are two kinds of immunoreactive cells called CD3 and CD8. CD3 is a common marker of all T cells. As a T cell antigen receptor and a signal transduction subunit, CD3 can transduce the antigen stimulation signal received by TCR into cells, activate T cells, and play an essential role in T cell immunity. The CD8-positive reaction cell is the immune cell closest to the intestinal cavity antigen in the entire intestinal mucosal immune system, and has an immune surveillance effect on bacterial and viral infections, and can specifically kill target cells directly [28].  Table 3 shows the enriched immunity for the small intestine terminal ileum.

The stomach tissue plays a significant role in local immune response and maintaining the stability of the internal environment [29]. And almost one-fourth of the gastrointestinal mucosa is a kind of lymphoid tissue, and its function is the same as that of the surrounding lymphoid tissue and the spleen. It has an important effect on the immune system.  Table 4 shows the enriched immunity for the stomach.

The phagocytosis, immunity, and secretion of lung macrophages are very active and have important defense functions. And the alveolar epithelium participates in gas exchange and constitutes a barrier to maintain lung function and also interacts with immune cells through its surface receptors and secretory products to maintain lung tissue homeostasis [30].  Table 5 shows the enriched immunity for the lung.

## 4. Discussion

Gene coexpression networks have been extensively studied recently due to its ability in finding key regulatory mechanisms and critical modules involved in a function. In this study, we constructed and compared gene coexpression networks and modules for 52 tissues and cell lines. There are a few interesting findings: (1) There are several modules existing in almost all coexpression networks, such as immune-related modules. (2) There are also some tissue-specific modules, for example, renal functions in the kidney. The results in this study might be important to explain tissue-common and tissue-specific diseases, since a few functions are highly associated with diseases.

We only conducted a simple module-based comparison on tissue networks. In the future, we will employ more network features by combining the graph theory with networks of tissue or modules, such as the distribution of network degree. Also, we can map disease genes to different tissues network to study the relationship between diseases and tissues based on disease-related information.

There are also some limitations of this study. For the selection of the power threshold, we selected 6 for all tissues and cell lines for a fair comparison, which will cause some coexpression networks not scare-free. It might be better to use different powers for different tissues. Besides, it takes a long time to construct the network because of the huge amounts of data. The efficiency of WGCNA method needs to be improved, especially in calculating the TOM similarity matrix. There are many methods to construct the gene coexpression network. However, a comprehensive comparison on these methods is not available. We will perform a comparison on a few popular methods and check if the results are consistent.

## 5. Conclusions

We constructed coexpression network and gene coexpression modules of 52 GTEx tissues and cell lines. We then compared the functions of modules and found that there are tissue common functions like immune-related functions and tissue-specific functions. A further analysis on the association of this function with diseases might be able to shed some light on the tissue specificity of diseases. We then studied the similarity among tissues in the perspective of gene coexpression networks and found that proximity tissues like different brain regions tend to have similar gene coexpression networks. Finally, the largest clique analysis was performed in the module network and 40 modules of the largest clique were found, which were related to immune functions. The result further demonstrated the generality of immune function in all tissues.

## Figures and Tables

**Table 1:** Enriched functions of genes for the spleen.

Category	Term	*p* value	Bonferroni	Benjamini	FDR
GOTERM_CC_FAT	GO:0005829~cytosol	7.52*E*-14	7.71*E*-11	1.93*E*-11	1.19*E*-10
GOTERM_CC_FAT	GO:0005783~endoplasmic reticulum	7.27*E*-11	7.46*E*-08	1.24*E*-08	1.15*E*-07
GOTERM_BP_FAT	GO:0010604~positive regulation of macromolecule metabolic process	1.35*E*-08	9.78*E*-05	4.89*E*-05	2.68*E*-05
GOTERM_CC_FAT	GO:0012505~endomembrane system	3.42*E*-08	3.51*E*-05	3.19*E*-06	5.42*E*-05
GOTERM_BP_FAT	GO:0022402~cell cycle process	3.61*E*-08	2.61*E*-04	6.53*E*-05	7.14*E*-05
GOTERM_BP_FAT	GO:0006357~regulation of transcription from RNA polymerase II promoter	3.71*E*-08	2.68*E*-04	5.36*E*-05	7.34*E*-05
GOTERM_CC_FAT	GO:0031090~organelle membrane	1.13*E*-07	1.16*E*-04	9.65*E*-06	1.79*E*-04
GOTERM_BP_FAT	GO:0010557~positive regulation of macromolecule biosynthetic process	1.53*E*-07	0.001105801	1.58*E*-04	3.03*E*-04
GOTERM_BP_FAT	GO:0009891~positive regulation of biosynthetic process	1.80*E*-07	0.001298936	1.62*E*-04	3.55*E*-04
GOTERM_BP_FAT	GO:0051173~positive regulation of nitrogen compound metabolic process	2.52*E*-07	0.001819388	1.66*E*-04	4.98*E*-04
GOTERM_BP_FAT	GO:0031328~positive regulation of cellular biosynthetic process	2.92*E*-07	0.002107957	1.76*E*-04	5.77*E*-04
GOTERM_MF_FAT	GO:0032553~ribonucleotide binding	3.34*E*-07	0.001058495	1.76*E*-04	6.06*E*-04
GOTERM_MF_FAT	GO:0032555~purine ribonucleotide binding	3.34*E*-07	0.001058495	1.76*E*-04	6.06*E*-04

**Table 2:** The immune GO result of the green module of the uterus.

Category	Term	*p* value	Fold enrichment	Bonferroni	Benjamini	FDR
GOTERM_BP_FAT	GO:0006955~immune response	6.56*E*-28	2.082379251	2.64*E*-24	2.64*E*-24	1.22*E*-24
GOTERM_BP_FAT	GO:0002684~positive regulation of immune system process	5.06*E*-15	2.392078274	2.05*E*-11	1.71*E*-12	9.49*E*-12
GOTERM_BP_FAT	GO:0002520~immune system development	4.23*E*-11	2.062734164	1.70*E*-07	8.96*E*-09	7.87*E*-08
GOTERM_BP_FAT	GO:0002703~regulation of leukocyte mediated immunity	1.32*E*-07	2.999901442	5.29*E*-04	1.82*E*-05	2.45*E*-04
GOTERM_BP_FAT	GO:0050778~positive regulation of immune response	4.46*E*-07	2.150120932	0.00179332	5.28*E*-05	8.30*E*-04
GOTERM_BP_FAT	GO:0002706~regulation of lymphocyte mediated immunity	7.04*E*-07	3.012246716	0.002827199	7.65*E*-05	0.001309
GOTERM_BP_FAT	GO:0002764~immune response-regulating signal transduction	1.52*E*-06	2.904666476	0.006106824	1.57*E*-04	0.002833
GOTERM_BP_FAT	GO:0002768~immune response-regulating cell surface receptor signaling pathway	2.17*E*-06	3.227407195	0.008678011	2.18*E*-04	0.00403

**Table 3:** The immune GO result of the green module of the small intestine terminal ileum.

Category	Term	*p* value	Fold enrichment	Bonferroni	Benjamini	FDR
GOTERM_BP_FAT	GO:0006955~immune response	6.56*E*-28	2.082379251	2.64*E*-24	2.64*E*-24	1.22*E*-24
GOTERM_BP_FAT	GO:0002684~positive regulation of immune system process	5.06*E*-15	2.392078274	2.05*E*-11	1.71*E*-12	9.49*E*-12
GOTERM_BP_FAT	GO:0002520~immune system development	4.23*E*-11	2.062734164	1.70*E*-07	8.96*E*-09	7.87*E*-08
GOTERM_BP_FAT	GO:0002703~regulation of leukocyte mediated immunity	1.32*E*-07	2.999901442	5.29*E*-04	1.82*E*-05	2.45*E*-04
GOTERM_BP_FAT	GO:0050778~positive regulation of immune response	4.46*E*-07	2.150120932	0.00179332	5.28*E*-05	8.30*E*-04
GOTERM_BP_FAT	GO:0002706~regulation of lymphocyte mediated immunity	7.04*E*-07	3.012246716	0.002827199	7.65*E*-05	0.001309
GOTERM_BP_FAT	GO:0002764~immune response regulating signal transduction	1.52*E*-06	2.904666476	0.006106824	1.57*E*-04	0.002833
GOTERM_BP_FAT	GO:0002768~immune response-regulating cell surface receptor signaling pathway	2.17*E*-06	3.227407195	0.008678011	2.18*E*-04	0.00403
GOTERM_BP_FAT	GO:0002683~negative regulation of immune system process	3.63*E*-06	2.449718715	0.014487533	3.39*E*-04	0.006748

**Table 4:** The immune GO result of the green module of the stomach.

Category	Term	*p* value	Fold enrichment	Bonferroni	Benjamini	FDR
GOTERM_BP_FAT	GO:0006955~immune response	3.99*E*-22	8.513043478	2.94*E*-19	2.94*E*-19	6.06*E*-19
GOTERM_BP_FAT	GO:0045087~innate immune response	1.10*E*-05	10.31884058	0.008077475	0.002699774	0.016689

**Table 5:** The immune GO result of the green yellow module of the lung.

Category	Term	*p* value	Fold enrichment	Bonferroni	Benjamini	FDR
GOTERM_BP_FAT	GO:0002684~positive regulation of immune system process	7.67*E*-08	3.085902662	2.22E-04	1.23*E*-05	1.38*E*-04
GOTERM_BP_FAT	GO:0006955~immune response	3.49*E*-07	2.025817914	0.001009227	4.81*E*-05	6.26*E*-04

## Data Availability

The data used in this study was downloaded from the GETx Official website (https://gtexportal.org).
